# Optical Micro/Nanofiber Enabled Multiaxial Force Sensor for Tactile Visualization and Human–Machine Interface

**DOI:** 10.1002/advs.202404343

**Published:** 2024-10-08

**Authors:** Yu Xie, Jing Pan, Longteng Yu, Hubiao Fang, Shaoliang Yu, Ning Zhou, Limin Tong, Lei Zhang

**Affiliations:** ^1^ Research Center for Frontier Fundamental Studies Zhejiang Lab Hangzhou 311100 China; ^2^ Research Center for Humanoid Sensing Zhejiang Lab Hangzhou 311100 China; ^3^ State Key Laboratory of Extreme Photonics and Instrumentation College of Optical Science and Engineering Zhejiang University Hangzhou 310027 China

**Keywords:** directional response, human–machine interface, micro/nanofiber (MNF), multiaxial force, optical sensor, tactile visualization

## Abstract

Tactile sensors with capability of multiaxial force perception play a vital role in robotics and human–machine interfaces. Flexible optical waveguide sensors have been an emerging paradigm in tactile sensing due to their high sensitivity, fast response, and antielectromagnetic interference. Herein, a flexible multiaxial force sensor enabled by U‐shaped optical micro/nanofibers (MNFs) is reported. The MNF is embedded within an elastomer film topped with a dome‐shaped protrusion. When the protrusion is subjected to vector forces, the embedded MNF undergoes anisotropic deformations, yielding time‐resolved variations in light transmission. Detection of both normal and shear forces is achieved with sensitivities reaching 50.7 dB N^−1^ (14% kPa^−1^) and 82.2 dB N^−1^ (21% kPa^−1^), respectively. Notably, the structural asymmetry of the MNF induces asymmetrical optical modes, granting the sensor directional responses to four‐directional shear forces. As proof‐of‐concept applications, tactile visualizations for texture and relief pattern recognition are realized with a spatial resolution of 160 µm. Moreover, a dual U‐shaped MNF configuration is demonstrated as a human–machine interface for cursor manipulation. This work represents a step towards advanced multiaxial tactile sensing.

## Graphical abstract

A multiaxial force sensor enabled by a U‐shaped optical micro/nanofiber (MNF) is developed. It offers special merits including high sensitivity, directional responsiveness, multifunctionality, excellent repeatability, and overload tolerance. The U‐shaped MNF is embedded within an elastomer film topped with a dome‐shaped protrusion, exhibiting the capability for detecting multiaxial forces. The sensor facilitates the development of both a high‐performance tactile visualization system and a multifunctional human–machine interface.

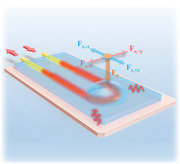

## 1 Introduction

Flexible tactile sensors are garnering widespread applications in areas such as intelligent robotics,^[^ ^1^, ^2^, ^3^, ^4^ ^]^ human–machine interactions,^[^ ^5^, ^6^, ^7^ ^]^ and wearable technologies.^[^ ^8^, ^9^, ^10^, ^11^ ^]^ Tactile sensors with multiaxial force perception are essential for both humans and robots to accurately identify and manipulate objects.^[^ ^12^, ^13^, ^14^, ^15^ ^]^ Typically, multiaxial force sensors (MAFSs) based on flexible electronics have advanced by utilizing stereo interleaved structure,^[^ ^16^ ^]^ interlocking microstructure (e.g., pyramid,^[^ ^17^ ^]^ hemisphere^[^ ^18^, ^19^ ^]^), cross‐laminated structure,^[^ ^20^ ^]^ and planar sensing array.^[^ ^21^, ^22^ ^]^ Despite their success in detecting normal and shear forces by monitoring distinct deformations in contact regions or relative movements between layers, issues like signal crosstalk and structural complexity still impede their wider use. Alternatively, anisotropic materials (e.g., Janus films^[^ ^23^, ^24^ ^]^) and structures^[^ ^25^, ^26^ ^]^ provide an effective scheme to construct both electronic and optical tactile sensors with the vector force detection capability.

In recent years, optical tactile sensors^[^ ^27^, ^28^, ^29^ ^]^ have attracted growing interest for their special merits including electrical safety, chemical inertness, high sensitivity, and flexibility. In 2022, Zhou et al. demonstrated a tactile sensor enabled by cross‐over waveguides that can distinguish between normal and shear forces.^[^ ^25^ ^]^ Leal‐Junior et al. demonstrated a spider web‐like optical waveguide sensor for force and orientation sensing.^[^ ^30^ ^]^ In 2023, Pan et al. proposed a knot‐inspired optical sensor for slip detection and friction measurements in dexterous robotic manipulation.^[^ ^26^ ^]^ However, these MAFSs still face challenges such as limited sensitivity, signal crosstalk, large size, and the complexity of calibration and decoupling algorithms. Optical micro/nanofibers (MNFs)^[^ ^31^, ^32^, ^33^ ^]^ with wavelength‐scale diameters offer minimal transmission loss (e.g., <0.05 dB cm^−1[^ ^34^ ^]^), high mechanical strength (e.g., tensile strength >5 GPa^[^ ^35^ ^]^), and compatibility with standard optical fibers. In particular, the strong evanescent fields outside the MNFs make them promising candidates for developing high‐performance tactile sensors that detect subtle pressures,^[^ ^36^, ^37^ ^]^ material hardness,^[^ ^38^ ^]^ and surface textures.^[^ ^39^ ^]^

In this study, we developed a U‐shaped MNF‐enabled MAFS with high sensitivity, directional responsiveness, multifunctionality, excellent repeatability, and overload tolerance. Experimentally, the U‐shaped MNF with a sub‐millimeter bending radius was embedded within a thin layer of low‐refractive‐index polydimethylsiloxane (PDMS) film, and topped with a dome‐shaped protrusion. Simulation results indicated that the protrusion transferred vector forces into anisotropic deformations of the waveguiding MNF, resulting in time‐resolved variations in light transmission. Detection of both normal and shear forces was achieved with sensitivities of 50.7 dB N^−1^ (14% kPa^−1^) and 82.2 dB N^−1^ (21% kPa^−1^), respectively. Notably, the asymmetric structure of MNF endowed the sensor with directional responses to shear forces from four different directions. Besides, we demonstrated the sensor's excellent repeatability and overload tolerance, both of which are essential for practical applications. To illustrate its potential applications, tactile visualizations for recognizing textures and relief patterns were achieved by sensor scanning. Moreover, a human–machine interface for the real‐time, multifunctional manipulation of a computer cursor was developed through a sensor comprising two U‐shaped MNFs.

## 2 Results

### 2.1 Concept and Principle of MNF‐Based MAFS

**Figure 1:**
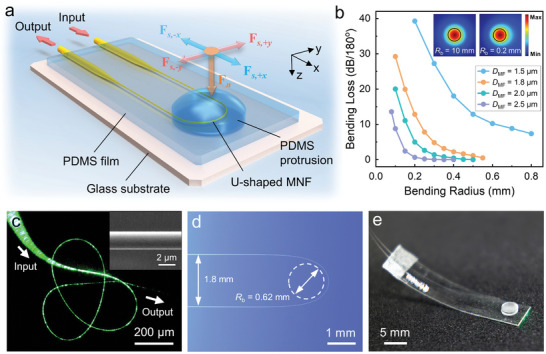
Flexible multiaxial force sensor based on a U‐shaped MNF. a) Schematic diagram of the sensor. The downward normal force is denoted by *F*_n_, and the shear forces in all four directions are denoted by *F*_s,+_ *_x_*, *F*_s,‐_ *_x_*, *F*_s,+_ *_y_*, and *F*_s,‐_ *_y_*, respectively. b) MNF bending loss as a function of bending radius (*R*_b_) and MNF diameter (*D*_MF_), insets: field distribution of the HE_11_ mode in MNFs with bending radius of 10 mm (left) and 0.2 mm (right), respectively. The MNF diameter *D*_MF_ = 2 µm, *n*_clad_ = 1.380, *n*_MF_ = 1.444. c) Dark‐field photograph of a wrapped MNF that transmitting green light, inset: scanning electron microscopy image of a 2 µm diameter MNF. d) Photograph of a U‐shaped MNF with a 2 µm diameter and a 0.62 mm bending radius. e) Photograph of as‐fabricated MNF‐based sensor.

**Figure** **1**a illustrates a schematic diagram of the MNF‐based MAFS. A SiO_2_ MNF is bent into a U‐shape and embedded within a low‐refractive‐index PDMS film. Above the film, a dome‐shaped PDMS protrusion is affixed to function as a pressure probe. Standard fibers are inherently connected to both ends of the MNF through tapered transition regions (i.e., the fiber taper), serving as the input and output channels for a 1550 nm laser source. Within the optical waveguide composed of an SiO_2_ core and PDMS cladding, light undergoes total internal reflection and propagates forward. Applying external forces to the protrusion causes the waveguiding MNF to deform, resulting in a transition between guided mode and radiated mode.^[^ ^32^ ^]^ Additionally, the photoelastic effect of the PDMS elastomer further improves the sensitivity. A reduced difference in the core/cladding refractive index leads to additional bending loss in the U‐shaped MNF (see details in the Supporting Information). Figure 1b presents the calculated bending loss of a U‐shaped MNF as a function of the bending radius (*R*_b_) at a 1550 nm wavelength. Thinner MNFs provide larger fractional evanescent fields around their periphery, making them more susceptible to radiation leakage upon external force stimuli. The insets in Figure 1b further depict the HE_11_ mode fields of a bent MNF with different bending radii (from 10 mm to 0.2 mm). The U‐shaped design disrupts the rotational symmetry of a straight MNF, significantly shifting the energy distribution of waveguiding mode toward the MNF's exterior (from 36.8% to 44.3%. *D*_MNF_ = 2 µm, *n*_clad_ = 1.38). This asymmetric distribution of mode fields enhances the MNF's sensitivity to force stimuli (refer to Figures S1 and S2, Supporting Information).^[^ ^32^ ^]^

Figure 1c shows an optical image of a curled SiO_2_ MNF fabricated by a taper‐drawing technique,^[^ ^40^ ^]^ which exhibits excellent flexibility with a bending radius as small as ≈100 µm. Besides, the excellent surface smoothness of MNF (inset in Figure 1c) ensures low‐loss light transmission and contributes to the long‐term stability of device. Figure 1d presents a U‐shaped MNF with a 2 µm diameter and a bending radius as small as 0.62 mm. By embedding the U‐shaped MNF into a thin layer of PDMS film, the packaged sensor (see Figure 1e) offers great adaptability, compactness, and resistance to contaminants like dust and water vapor.

**Figure 2:**
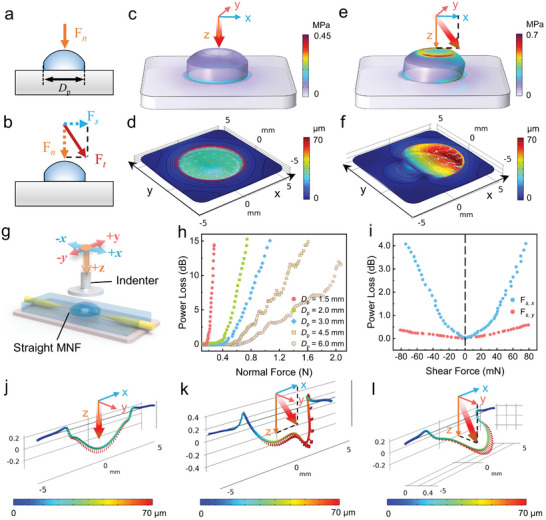
Structural mechanics simulations and experimental studies of PDMS protrusion and straight‐MNF based sensor. a,b) Schematic illustrations of forces applied to a flexible protrusion with a diameter (*D*_p_) include: a) a downward normal force *F*_n_ and b) an arbitrary directional force *F*_t_. *F*_s_ represents the shear force. c–f) Structural mechanics simulations to investigate mechanical behaviors of the dome‐shaped PDMS protrusion (*D*_p_ = 4 mm) and PDMS film: c,d) *F*_n_ = 2.0 N; e,f) *F*_n_ = 2.0 N, *F*_s,_ *_x_* = 1.0 N. g) A protrusion‐integrated sensor with a straight MNF embedded within a PDMS film. A hard indenter was employed to provide multiaxial forces. h) Normal force responses with protrusions of various diameters as pressure probes. i) Shear force responses including *F*_s_ *_, ±x_* and *F*_s_ *_, ±y_*. *D*_p_ = 4 mm. j–l) Structural mechanics simulations of the sensor under various forces with *D*_p_ = 4 mm. j) *F*_n_ = 2.0 N, k) *F*_n_ = 2.0 N, *F*_s_ *_,+x_* = 1.0 N, and l) *F*_n_ = 2.0 N, *F*_s_ *_,+y_* = 1.0 N.

When the PDMS protrusion is subjected to either a vertically downward normal force (*F*_n_. **Figure** **2**a) or an oblique force (vectorially decomposed into F_n_ and the shear force *F*_s_. Figure 2b), the PDMS protrusion experiences specific deformations that reflect the characteristics of applied forces. The structural mechanics simulations in Figure 2c,d illustrate that the protrusion together with the underlying film undergoes a rotationally symmetric deformation and spreads radially upon the normal force (*F*_n_ = 2.0 N). However, an oblique force (*F*_n_ = 2.0 N and an *x*‐directional shear force *F*_s, x_ = 1.0 N) will cause asymmetric compression and shear deformation to them (Figure 2e,f). It can be observed that the film develops a pronounced depression along the force direction, while the opposite side essentially returns to its unstrained state.

To further investigate the role of protrusions, dome‐shaped PDMS protrusions were positioned atop a straight MNF that embedded within a PDMS film (see Figure 2g). Multiaxial force response tests (Figure 2h,i) and corresponding structural mechanics simulations (Figure 2j–l) were both carried out. Figure 2h shows that a protrusion with a larger diameter provided lower sensitivity to normal forces due to the reduced pressure. Notably, the sensor displayed a threshold phenomenon in response to normal forces,^[^ ^38^ ^]^ which was due to the elastomer's cushioning effect and the straight MNF's insensitivity to bending over large radii under weak pressure. As Figure 2j indicates, the straight MNF undergoes a downward and large‐radius bending upon normal forces, generating a relatively small bending loss accordingly. When it comes to shear forces (Figure 2i, *D*_p_ = 4.0 mm, *F*_n_ = 1.5 N), the MNF showed expected symmetrical responses in both the ±*x* and ±*y* directions, with average sensitivities of 59.2 ± 2.4 and 5.9 ± 1.5 dB N^−1^, respectively. The significant difference in sensitivity between *F*_s, ±_ *_x_* and *F*_s, ±_ *_y_* results from more pronounced shear deformation along the axial direction (cf., Figure 2k,l). In summary, the protrusion acts as a flexible mechanical interface, converting various types of forces into spatially distributed deformations within the elastic film, facilitating the development of feature‐rich MAFSs.

### 2.2 Characterization of the MAFS

**Figure 3:**
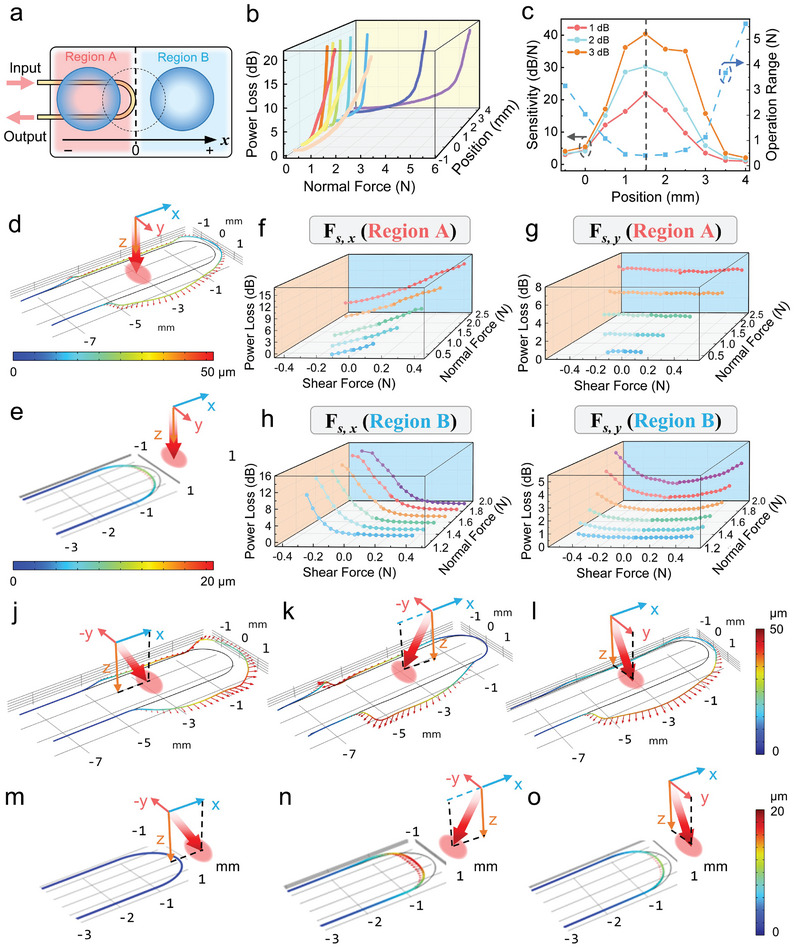
Multiaxial force sensing based on a U‐shaped MNF. a) Schematic illustration of adjusting the protrusion's position along the *x*‐axis in relation to the U‐shaped MNF. The rightmost bending point of MNF is designated as the origin. b) Evolution of the response to normal force as a function of protrusion position. The examined response depth is up to 20 dB. c) Sensitivity and operation range in relation to the protrusion position. d,e) Structural mechanics simulations of the sensor with the protrusion located at d) −3.0 mm and e) +3.0 mm, respectively. The applied forces were *F*_n_ = 2.0 N, *F*_s_ *_, x_* = 0, and *F*_s_ *_, y_* = 0. f,g) Response to shear forces of f) *F*_s_ *_, x_* and g) *F*_s, y_ with the protrusion in region “A” (*x* = −3.0 mm). h,i) Response to shear forces of h) *F*_s_ *_,x_* and i) *F*_s_ *_,y_* with the protrusion in region “B” (*x* = +3.0 mm). j–o) Structural mechanics simulations of the sensor with the protrusion at j–l) *x* = ‐3 mm and m–o) *x* = +3 mm. The applied forces were: j,m) *F*_n_ = 2.0 N, *F*_s_ *_, x_* = 0.4 N, k,n) *F*_n_ = 2.0 N, *F*_s_ *_, x_* = −0.4 N, l,o) *F*_n_ = 2.0 N, *F*_s, y_ = 0.4 N.

To explore the feasibility of breaking structural symmetry for realizing anisotropic responses in force detection, we curled the straight MNF into a U‐shape with a sub‐millimeter bending radius. Both the axial symmetry about the *y* axis and the rotational symmetry along the fiber axis were disrupted (refer to Figure 1a). This minimal bending radius of MNF enhances its sensitivity to external forces while also miniaturizes the device. The force responses of the MAFS can be flexibly regulated by repositioning the protrusion relative to MNF, as illustrated in **Figure** **3**a. We evaluated the normal force responses across a dynamic range up to 20 dB, as displayed in Figure 3b,c. The sensor exhibited distinct response characteristics across three regions along the *x*‐axis (i.e., *x* ≤ 0.5 mm, 0.5 mm < *x* <2.5 mm, and *x* ≥ 2.5 mm). For example, positioning the protrusion at *x* = −0.5 mm yielded low sensitivity to normal forces within an operation range of approximately 3 N. At *x* = 1.5 mm, the sensor reached an ultrahigh sensitivity of up to 50.7 dB N^−1^ (equivalent to a pressure sensitivity of 14% kPa^−1^), while the operation range narrowed to 0.4 N accordingly. Based on the noise floor of employed photodetector, the sensor's resolution for detecting normal forces was determined to be 120 µN (equating to a pressure of ≈9.5 Pa). At *x* = 3.5 mm, we observed a wide range of force responses, with sensitivities ranging from low (≈0.3 dB N^−1^) to high (≈29.6 dB N^−1^) for forces between 0–1.0 and 3.3–3.7 N, respectively. We conducted structural mechanics simulations at various protrusion positions to clarify the mechanisms behind these specific responses. As depicted in Figure 3d (*x* = −3.0 mm), the normal force directly deforms the fiber taper downwards, while the U‐shaped MNF experiences pronounced bending by the diffusive pressure from the PDMS cladding. Similarly, the normal force directly impacts the MNF's bending area at 0.5 mm < *x* <2.5 mm, causing considerable bending loss and resulting in the greatest sensitivity (which is called a highly‐sensitive mode). Furthermore, positioning the protrusion farther from the MNF (e.g., *x* = 3.0 mm, see Figure 3e) shifts the sensor to operate in a high‐threshold mode. The cushioning effect of the PDMS elastomer mitigates the force, yielding a diminished response from the MNF. A greater force deepens the compression of cladding, deforming the adjacent MNF and leading to lower sensitivity.

In investigating the response of sensor to shear forces, we examined two specific positions within regions “A” and “B” (at *x* = ‐3 mm and *x* = +3 mm, respectively, see Figure 3a). The experimental results and corresponding simulations are presented in Figure 3f–i,j–o, respectively. As depicted in Figure 3f, with the protrusion at *x* = ‐3 mm, the sensor exhibited directional response to shear forces along the *x*‐axis (denoted by *F*_s,_ *_‐x_* and *F_s_*_,_ *_+x_*, respectively). Similar responses (9.3–15.2 dB N^−1^) to bidirectional shear forces were observed, albeit with completely opposite response trends. Under *F_s_*_, +_ *_x_*, the nonuniform deformation of the PDMS cladding led to additional extrusion in both the fiber taper and the U‐shaped MNF (cf., Figure 3d,j), resulting in a decreased transmission. Conversely, *F*_s_ *_, ‐x_* increased bending within the fiber taper, reverting the U‐shaped region from extruded to uncompressed state (cf., Figure 3d,k), resulting in an increased transmission. Interestingly, the sensor was insensitive to shear forces along the *y*‐axis (denoted as *F*_s_ *_, ‐y_* and *F*_s_ *_, +y_*, respectively), with RMS deviations below 0.12 dB (Figure 3g). This insusceptibility was attributed to the MNF's axial symmetry about the *x* axis. Figure 3l illustrates that exposure to *F*_s,_ *_+y_* or *F*_s,_ *_‐y_* caused symmetric bending changes in the two fiber tapers, ultimately neutralizing the response to *F*_s_ *_, ±y_*.

At *x* = +3 mm of region “B” (Figure 3h,i), the U‐shaped MNF was predominantly subjected to pulling or squeezing by the cladding. The response to shear forces *F*_s_ *_, ±x_* differed markedly from that in region “A” (Figure 3f), offering an ultrahigh sensitivity of up to 82.2 dB N^−1^ (equivalent to 21% kPa^−1^) for *F*_s_ *_, ‐x_*. The forces *F*_s_ *_, +x_* and *F*_s_ *_, ‐x_* caused the MNF to revert to its original state (Figure 3m) and to undergo additional compressive deformation (Figure 3n), yielding time‐resolved variations in MNF transmission, respectively. The MNF's axial symmetry around the *x* axis also led to symmetrical responses to *F*_s_ *_, ±y_* (≈2.0 dB N^−1^), mainly from minor strain in the U‐shaped region (Figure 3o). Consequently, by integrating a dome‐shaped pressure probe with an asymmetrically designed MNF, we developed a directionally responsive MAFS that can be flexibly customized. It offers high sensitivities to multiaxial forces that significantly exceed those of other recently reported optical MAFSs^[^ ^25^, ^26^, ^41^, ^42^ ^]^ (refer to Table S1 in the Supporting Information). The innovative design not only minimizes signal crosstalk from various forces, but also lessens the sensor's reliance on multiple sensing units and algorithms.

**Figure 4:**
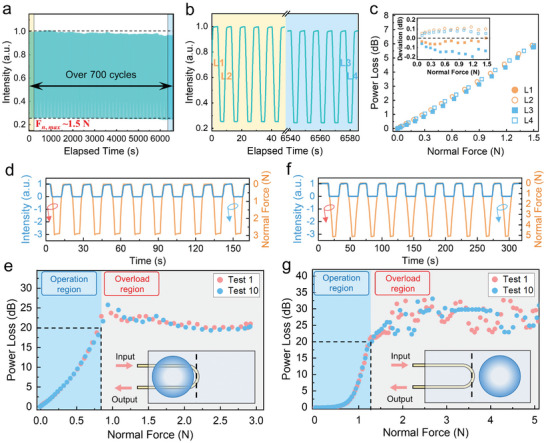
Repeatability and overload tolerance of U‐shaped MNF sensor. a) Normalized transmission curve of the sensor when subjected to more than 700 cycles of normal forces. The maximum normal force (*F*_n,max_) applied was approximately 1.5 N. b) Zoomed‐in view of the transmission curve of initial and final press periods from (a). c) Response curves extracted from the lines marked “L1‐4″ in (b), including the downward pressing (L1 and L3) and upward retraction (L2 and L4) phases. Inset: root mean square (RMS) deviations of response curves. d–g) Cyclic overload tests on the sensor with response depths reaching up to 20 dB. The protrusion was mounted in (d,e) region “A” and (f,g) region “B”, respectively. Here, e) and g) present the response curves extracted from (d) and (f), respectively. The operation range was defined as the normal force range that resulted in response depths within 20 dB, while responses beyond this threshold indicating the overload region.

To evaluate the response repeatability of our MAFS, an automated test was employed to test its optical response to normal force. **Figure** **4**a shows minimal drift in transmission baseline while being pressed over 700 cycles. Figure 4b presents a detailed comparison of normalized transmission between the initial and final cycles, from which the pressing (“L1” and “L3”) and retraction (“L2” and “L4”) phases were further extracted and presented in Figure 4c. This reveals consistent sensor responsiveness with RMS deviations within ±0.2 dB (inset of Figure 4c). The overload capacity of the sensor was also examined through cyclic testing, where it endured response depths surpassing 20 dB in regions “A” (Figure 4d,e) and “B” (Figure 4f,g). The consistency of response curves from the initial and final cycles suggests that the sensor could withstand loads approximately 3.8 times its rated operating range (up to 3.0 N from 0–0.8 N, see Figure 4e), and 3.9 times (up to 5.1 N from 0–1.3 N, see Figure 4g) when the protrusion was in region “B.” Increasing the distance between the protrusion and U‐shaped MNF further enhances the sensor's overload resistance to nearly 10 N, equivalent to a pressure of 0.8 MPa (refer to Figure S9, Supporting Information). In conclusion, benefiting from its embedding in a low‐refractive‐index elastic film, the MNF‐based MAFS not only offers remarkable repeatability but also superior overload tolerance. These attributes are crucial for tactile sensing applications requiring both high accuracy and durability.

### 2.3 Tactile Visualization

**Figure 5:**
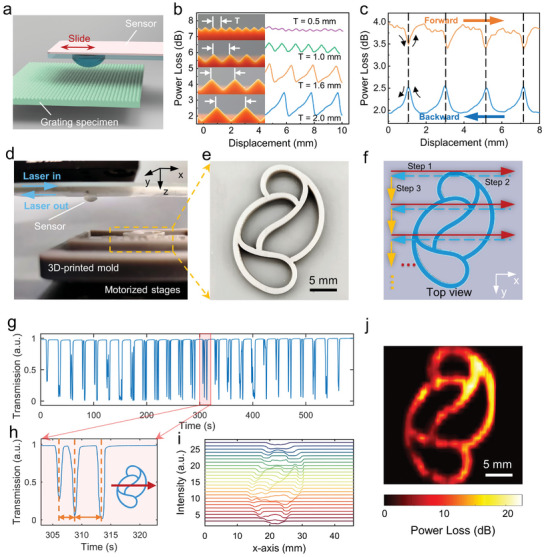
Tactile visualization for texture and relief pattern recognition. a) Schematic illustration of texture recognition by sliding the sensor over a grating. The sensor was fabricated using a single U‐shaped MNF. b) Time‐resolved transmission curves related to the sensor's displacement over gratings with varied periods. Insets: gratings with varying periods and undulations that were used for texture recognition. The grating with a period of *T* = 0.5 mm had undulations as fine as 160 µm. c) Transmission curves related to the sensor's displacement while sliding forwards and backwards across a 2 mm period grating. d) Photograph of the experimental setup used for relief pattern recognition. e) Photograph of a 3D‐printed specimen emblazoned with a relief pattern. f) Scanning motions of the motorized stage in the horizontal plane. g) Continuous transmission signals acquired from sensor scanning. h) Enlarged view of transmission curve from (g), as the sensor passed over the region shown in the right inset. i) Position‐dependent segmented curves derived from (g). j) Reconstructed image of the relief pattern.

To verify the practicality and versatility of our MAFS, we initially applied it to texture recognition in its highly sensitive mode (i.e., the protrusion was located at region “A”). Due to the inherently localized characteristic of textures, the high sensitivity of sensor enables the precise detection of subtle variations in texture patterns. **Figure** **5**a schematically illustrates the sensor affixed to a glass substrate and inverted horizontally, sliding across textured specimens via the protrusion. The specimens shown in Figure 5b consisted of periodic gratings with various periods (*T*, ranging from 0.5 to 2.0 mm) and height undulations (the smallest being 160 µm). The mechanical interaction between the protrusion and localized ridges resulted in time‐resolved fluctuations in sensor transmission. The sensor distinctly detected undulations as fine as 160 µm, indicating a spatial resolution comparable to mechanoreceptors in human fingertips.^[^ ^43^ ^]^ Also, the notch depths of gratings were reflected in the fluctuation amplitude of transmission signals (see Figure S10, Supporting Information). Figure 5c highlights the sensor to distinguish between forward and backward sliding motions through its directional sensing capabilities, yielding two transmission evolution curves in opposite directions. In terms of classifying various textures, as depicted in Figure S11 (Supporting Information), when the sensor slid over surfaces of corduroy fabric, tweed fabric, and plank, distinct transmission variations were generated accordingly. The periodicity and amplitude of the transmission fluctuations reflected the texture characteristics of material surfaces. The integration of machine learning algorithms is anticipated to enable precise and automated texture recognition tasks. Hence, unlike conventional sensors that can only recognize texture fluctuations, the single MNF‐based flexible sensor offers advantages for simultaneously identifying surface texture and sliding direction, demonstrating promise for use in robotic grasping and manipulation.^[^ ^17^, ^44^ ^]^

Building on the success with 1D textures, we extended our investigation to 2D tactile visualization with a sensor‐based scanning method. Figure 5d shows the experimental setup, where the sensor was positioned beneath a bracket, with a 3D‐printed relief pattern (with a linewidth of 1 mm and a height of 2 mm, see Figure 5e) secured on a motorized stage below. Figure 5f indicates the programmed horizontal stepping movements of the stage, which facilitated a 2D scanning operation over the specimen by the sensor. This approach generated a continuous stream of transmission signals (Figure 5g). A detailed view in Figure 5h reveals three distinct dips during the protrusion scanning across the relief pattern. By converting these time‐sequential signals into segmented and position‐dependent curves, we could accurately delineate the pattern's structural features (Figure 5i). The final reconstructed image for tactile visualization is presented in Figure 5j, which is highly consistent with the features of the original specimen.

Notably, the sensor generated increased transmission loss as it traversed horizontally over a slope (refer to Figure S12, Supporting Information), indicating the feasibility of quantifying surface undulation height. Besides, the spatial resolution of tactile sensing is primarily constrained by the protrusion's rounded‐top radius. A decrease in radius indicates a reduced contact area with the localized texture features, enabling the probe to more readily discern and discriminate subtle textural variances. Consequently, appropriately reducing the radius is an effective strategy to enhance spatial resolution for such visualization tasks.

### 2.4 Human–Machine Interface

**Figure 6:**
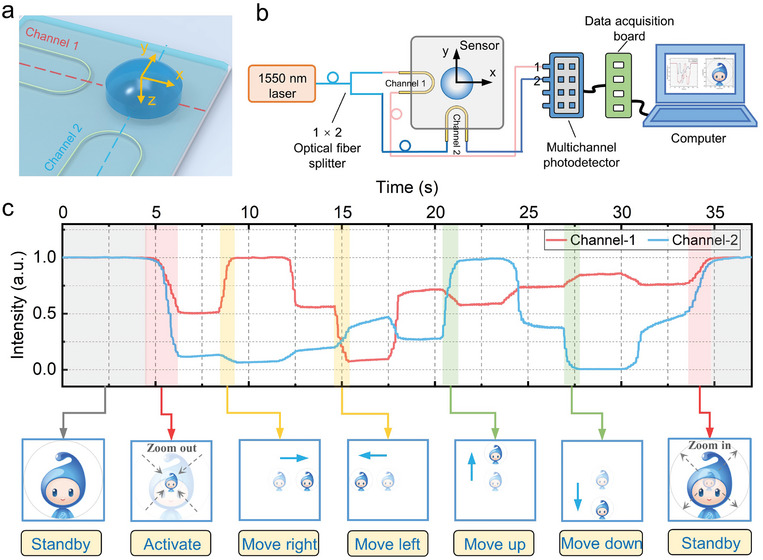
Human–machine interface for real‐time multifunctional manipulation of a computer cursor. a) Schematic illustration of the dual‐MNF sensor, which integrates two orthogonally arranged U‐shaped MNFs for directional recognition. b) Schematic illustration of the experimental setup, where the two MNFs function as independent channels. c) Time‐resolved transmission curves from the dual‐channel sensor during multifunctional cursor manipulation. The lower insets depict the states of “XiaoZhi” cursor (from left to right): standby, zoom‐out activation, four‐directional movements, and zoom‐in to standby state.

Furthermore, we developed a human–machine interface for virtual manipulation by utilizing the sensor's directional responses. Compared to demonstrations mentioned above, this interface employed a tactile sensor integrated with two U‐shaped MNFs that are orthogonally arranged, operating in the high‐threshold mode (**Figure** **6**a). The sensor with a dual‐MNF configuration enabled the detection of four‐directional shear forces, not only minimizing signal crosstalk but also simplifying user interaction logic in the human–machine interface, and reducing the complexity of data processing algorithms. The cursor movements were triggered by the signals of directional shear forces applied to the protrusion. Once a directional shear force was detected (i.e., transmission variation beyond the defined threshold), the cursor moved in the corresponding direction until the force was unloaded. Therefore, intuitive mapping relations between the signals and directional movements (i.e., ±*x*, ±*y*) of the computer cursor were established. The sensor's response threshold to normal forces prevents false signals, thus avoiding unintentional cursor movements when in a standby mode. Figure 6b depicts the experimental setup, a 1550 nm laser was split by a 1 × 2 optical fiber splitter, coupling light into two channels of the sensor (i.e., the two U‐shaped MNFs), with the outputs connected to a multichannel photodetector. A high‐speed data acquisition board collected signals from the photodetector and transmitted them to a computer for real‐time analysis. The sampling rate of the data acquisition board was set to 200 Hz per channel, and the image refresh rate was maintained approximately 100 fps. As a demonstration shown in Figure 6c (refer also to Movie S1, Supporting Information), a gentle force left the transmissions of sensor (*T*_1,2_ ≈ 1) unaffected, maintaining a large “XiaoZhi” image on the display to indicate a standby state. Upon a normal force exceeded the threshold (*T*_1,2_ < 50%), the “XiaoZhi” image shrunk into a cursor to activate the running state. Applying directional shear forces allowed the cursor to move in corresponding directions. Conversely, a reduction in force below the threshold (*T*_1,2_ > 50%) returned the cursor to its initial standby state. Integrating advanced feature recognition algorithms and artificial intelligence algorithms is expected to significantly enhance the functionalities of our sensor in human–machine interface applications.

## 3 Conclusion

In conclusion, we proposed an innovative strategy utilizing an asymmetric configuration of optical waveguides to achieve directional responses. This methodology is highly versatile and can be extended to other optical fibers and flexible waveguides. The developed U‐shaped MNF‐enabled MAFS is characterized by high sensitivity, directional responsiveness, and multifunctionality. By utilizing structural mechanics simulations, we explored the micromechanical behaviors of the dome‐shaped protrusion and the MNF embedded within an elastomer film. Besides, we experimentally demonstrated a MAFS integrated with a U‐shaped MNF, which was highly sensitive to both normal and shear forces. Notably, the asymmetric structural design of the MNF endowed the sensor with directional responses to shear forces. This advancement enables the creation of both a high‐performance tactile visualization system and an innovative multifunctional human–machine interface. The optical sensor holds great potential for diverse applications in robotic haptics, human–machine interaction, and wearable technologies.

## 4 Experimental Section

### Simulations

Optical simulations of calculating bending losses and fields distributions of MNFs were conducted by using MODE solutions and COMSOL Multiphysics, respectively (see details in Figures S1 and S2, Supporting Information). Structural mechanics simulations were conducted by COMSOL Multiphysics software. The materials for the protrusion and film were assumed to be PDMS (Young's modulus: 2.2 MPa. Poisson's ratio: 0.47. Density: 970 kg m^−3^). The diameter of dome‐shaped protrusion was 4.0 mm. The upper and lower thicknesses of PDMS film were 300 and 500 µm, respectively. During the simulations, a flat‐bottomed indenter that similar to the one used in experimental tests was employed to apply forces to the protrusion. Downward normal forces and shear forces in four directions were applied through the relative displacements of the indenter against the top surface of the protrusion. There was a monotonic correlation between the displacement with the applied force (see details in Figure S3, Supporting Information).

### Fabrication of MNF

The SiO_2_ MNFs were fabricated using a taper‐drawing technique (see Figure S5, Supporting Information).^[^ ^40^ ^]^ Commercially available single‐mode SiO_2_ fibers (Corning SMF‐28e) were heated to a temperature exceeding the softening point of silica glass using a hydrogen flame. Subsequently, under the control of high‐precision translation stages, the fibers were stretched bi‐directionally at a constant speed of 0.1 mm s^−1^ on both sides. A 785 nm laser source coupled with an optical power meter was connected to each end of the fiber to monitor its transmittance throughout the taper‐drawing process. By monitoring the abrupt drop in transmission due to the cutoff of high‐order modes, the MNF diameter could be precisely controlled.

U‐shaped MNFs were reproducibly fabricated using a parallel capillary constraint method (see details in Figure S6a, Supporting Information). U‐shaped MNFs with bending radii ranging from ≈100 µm to several millimeters could be fabricated within a few minutes, exhibiting a bending radius deviation of less than 10%. A specially designed mold comprised two parallel glass capillaries, spaced a distance (*L*_0_) apart, determining the fiber ends’ separation and consequently the MNF's bending radius (*R*_b_ ≈ *L*_0_/2). The inner diameter of capillaries was designed to be slightly larger than the diameter of standard optical fiber, allowing the fiber to pass through smoothly and steadily. A thin polyethylene terephthalate (PET) film was mounted on the mold to support and stabilize the fiber. After the optical fiber being threaded through the capillaries, it was bonded to the PET film using UV‐curable adhesive. Upon removing the mold, a U‐shaped MNF securely attached to a PET film was quickly obtained.

To achieve high sensitivity of the MAFS to external forces, minimize multimode interference, and simplify device fabrication, U‐shaped MNFs with a diameter of 2 µm and a bending radius as small as ≈600 µm were fabricated for further investigation.

### Fabrication of Sensor

The sensor consisted of either a straight MNF or a U‐shaped MNF was fabricated using a bottom‐up approach (see Figure S6b, Supporting Information).^[^ ^37^ ^]^ Initially, a PDMS solution was drop‐coated onto a glass substrate to form a flexible base layer approximately 500 µm thick. The MNF was then placed onto this PDMS layer, followed by another layer of PDMS solution to create a ≈300 µm thick upper cladding. Additionally, the protrusion was designed with a rough topography on its curved surface to enhance grip, with a flat and smooth underside for better adherence to the underlying PDMS film. PDMS protrusions a few millimeters in diameter were crafted using 3D‐printed molds. The protrusions were precisely transferred and positioned by micromanipulation under a stereo microscope. The PDMS solution was prepared by mixing prepolymers with curing agents in a 10:1 ratio. The PDMS films were solidified by baking at 40 °C for 10 h, and the protrusions by baking at 80 °C for 1 h. Finally, to obtain a freestanding and flexible photonic device, anhydrous ethanol was introduced to gently separate the sensor from the glass substrate. For the demonstration experiments, a thin layer of PDMS solution was scraped between the protrusion and PDMS cladding to ensure a fully integrated connection.

### Characterization

A commercially available laser diode (CLD1010LP, Thorlabs) and a multichannel photodetector (PD10A‐200M‐4ch, Guilin Guangyi) were utilized to characterize the MAFSs. Significant bending or stretching in proximity to the sensor's sensitive region can induce additional perturbations in the transmission characteristics of the MNF, thereby impeding the accurate measurement of multiaxial forces. The sensitive region covers several square millimeters and is ideally affixed to a substrate that is minimally susceptibility to undesired stretching or bending. In practical application scenarios where the sensor undergoes deformation, it is crucial to perform force calibration prior to employment.

In PDMS‐film‐embedded MNF sensors (see Figure S7a, Supporting Information), the lack of a specific stressed area leads to sensitivity variations. As seen in Figure S7b (Supporting Information), a smaller area under stress leads to sharper bending in the straight MNF, making the sensor more responsive to normal forces. For example, under a 5 mm diameter indenter, the average sensitivity reached approximately 18.5 dB N^−1^, whereas it decreased to only 1.6 dB N^−1^ when using an 8 mm diameter indenter. Therefore, the protrusion as a pressure probe was employed to quantify the response of sensor to external forces.

Figure S7c (Supporting Information) shows the custom‐built experimental setup designed to characterize the sensor's response to multiaxial forces, featuring a commercial six‐axis force sensor (Sunrise Instruments, M3815A) and motorized stages. A laterally configurated imaging system was utilized to monitor the spacing between the indenter and the PDMS protrusion in real time. Figure S7d (Supporting Information) presents optical micrographs that illustrate the significant elastic deformation of the PDMS protrusion when force is applied through the indenter. Under transient pressures, the high‐damping PDMS elastomer acted as an external cushion, potentially impeding the embedded MNF from delivering reliable and precise optical signals. As shown in Figure S7e (Supporting Information), the sensor maintained consistent responses when the velocity of normal force was kept below 5.9 N s^−1^. In contrast, increasing the force velocity to 9.4 N s^−1^ resulted in obvious discrepancies in the sensor's response (≤0.5 dB, as indicated in the inset of Figure S7e, Supporting Information), particularly with stronger normal forces. Consequently, to ensure consistent sensor responses, the normal force velocity was maintained at or below 5.9 N s^−1^ during the experiments reported in this manuscript. Meanwhile, it is important to highlight that the photoelastic effect of the PDMS film alters the cladding refractive index when compressed, which directly impacts the modal coupling conditions of the waveguiding MNF. Consequently, such alterations might result in a nonmonotonic force response of the sensor, thereby potentially narrowing the operation range.^[^ ^45^ ^]^ Further investigations were conducted and shown in Figure S8 (Supporting Information).

## Conflict of Interest

The authors declare no conflict of interest.

## Supporting information

Supporting Information

Supplemental Movie 1

## Data Availability

The data that support the findings of this study are available in the Supporting Information of this article.
