# Supplementary material for: Optical Micro/Nanofiber Enabled Multiaxial Force Sensor for Tactile Visualization and Human–Machine Interface
Source: Adv Sci (Weinh). 2024 Oct 8;11(45):2404343. doi: 10.1002/advs.202404343 (PMC11615745; doi:10.1002/advs.202404343)
Supplement: Supplementary file 1 — Supporting Information [file ADVS-11-2404343-s001.docx]

**Supporting Information**

**Optical Micro/Nanofiber Enabled Multiaxial Force Sensor for Tactile Visualization and Human-Machine Interface**

*Yu Xie*, *Jing Pan**, *Longteng Yu*, *Hubiao Fang, Shaoliang Yu, Ning Zhou*, *Limin Tong*, *and* *Lei Zhang**

# Optical simulations of U-shaped MNF

As illustrated in Figure S1a, radiation leakage occurs during light propagating through the U-shaped region of the micro/nanofiber (MNF). The evanescent fields outside the MNF is significantly influenced by several optical waveguiding parameters, including the MNF diameter (*D*_MF_), bending radius (*R*_b_), refractive indices of the MNF (*n*_MF_) and cladding (*n*_clad_), and the guided wavelength (*λ*)^[1]^. As shown in Figures S1b,c, there is more energy exists outside the MNF with a smaller *R*_b_. Also, MNFs with smaller diameters or slighter differences in core/cladding refractive indices provide larger fractional evanescent fields around their periphery, rendering them more susceptible to radiation leakage upon external force stimuli. For example, an MNF with a diameter of 1.5 μm disperses more than 83% of its energy outside the surface (Figure S1b. *n*_MF_ = 1.444, *n*_clad_ = 1.380, *λ* = 1550 nm, *R*_b_ = 0.1 mm).

The bending loss of an MNF is closely related to *n*_clad_^[2]^. As depicted in Figure S1d, the increase in *n*_clad_ causes greater bending loss. Insets in Figure S1d show the HE_11_ mode for an MNF with a 2 μm diameter and a bending radius of 1.0 mm. When *n*_clad_ increases from 1.38 to 1.41, the energy distribution substantially shifts towards the outside of the fiber (from 36.8% to 68.5%). Besides, as the sharpness of the bend increases, so does the susceptibility to significant bending losses. Figures S2a-c illustrate that within the bending region, the light field becomes asymmetric and progressively shifts outward, leading to increased radiation leakage. A higher cladding refractive index further contributes to this asymmetry in field distribution (see Figures S2c-e). Compared to elastomer optical waveguides with millimeter-sized cross sections, the wavelength-scale MNF offers a significantly larger fraction of evanescent waves, thereby providing ultra-high sensitivity to external stimuli and showing immense potential for high-performance optical sensing applications.


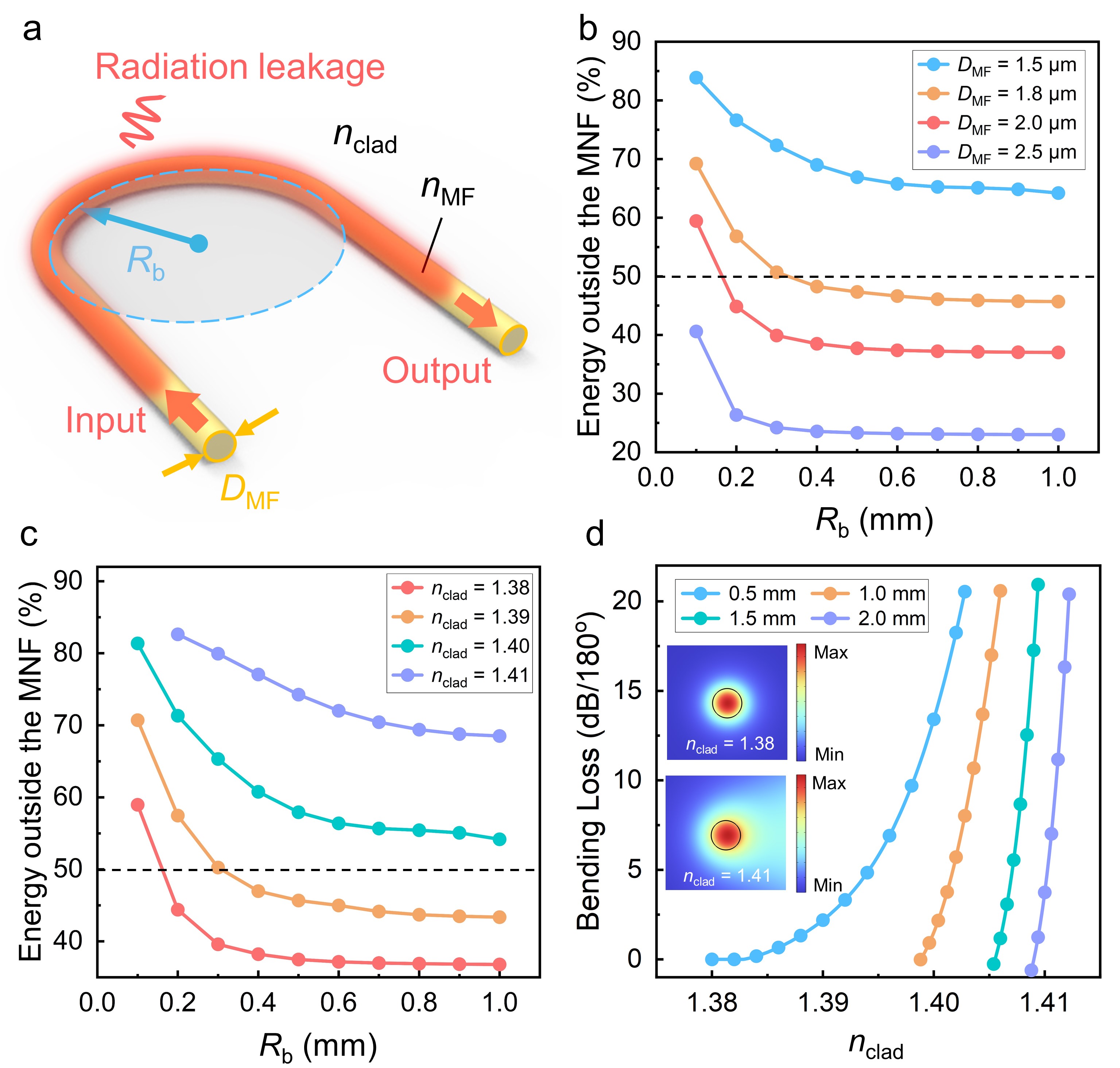


**Figure S1**. Optical simulations of the U-shaped MNF. (a) Schematic illustration of light propagation through the bent MNF. *D*_MF_, MNF diameter. *R*_b_, bending radius. *n*_MF_, MNF refractive index*. n*_clad_, cladding refractive index; (b) Fractional energy outside the MNF as a function of bending radius for various *D*_MF_, with *n*_MF_ = 1.444 and *n*_clad_ = 1.380; (c) Fractional energy outside the MNF as a function of bending radius for various *n*_clad_, with *n*_MF_ = 1.444 and *D*_MF_ = 2.0 μm; (d) Bending loss of the MNF as a function of *n*_clad_ for various *R*_b_, with *n*_MF_ = 1.444 and *D*_MF_ = 2.0 μm. The guided light wavelength is *λ* = 1550 nm.

**
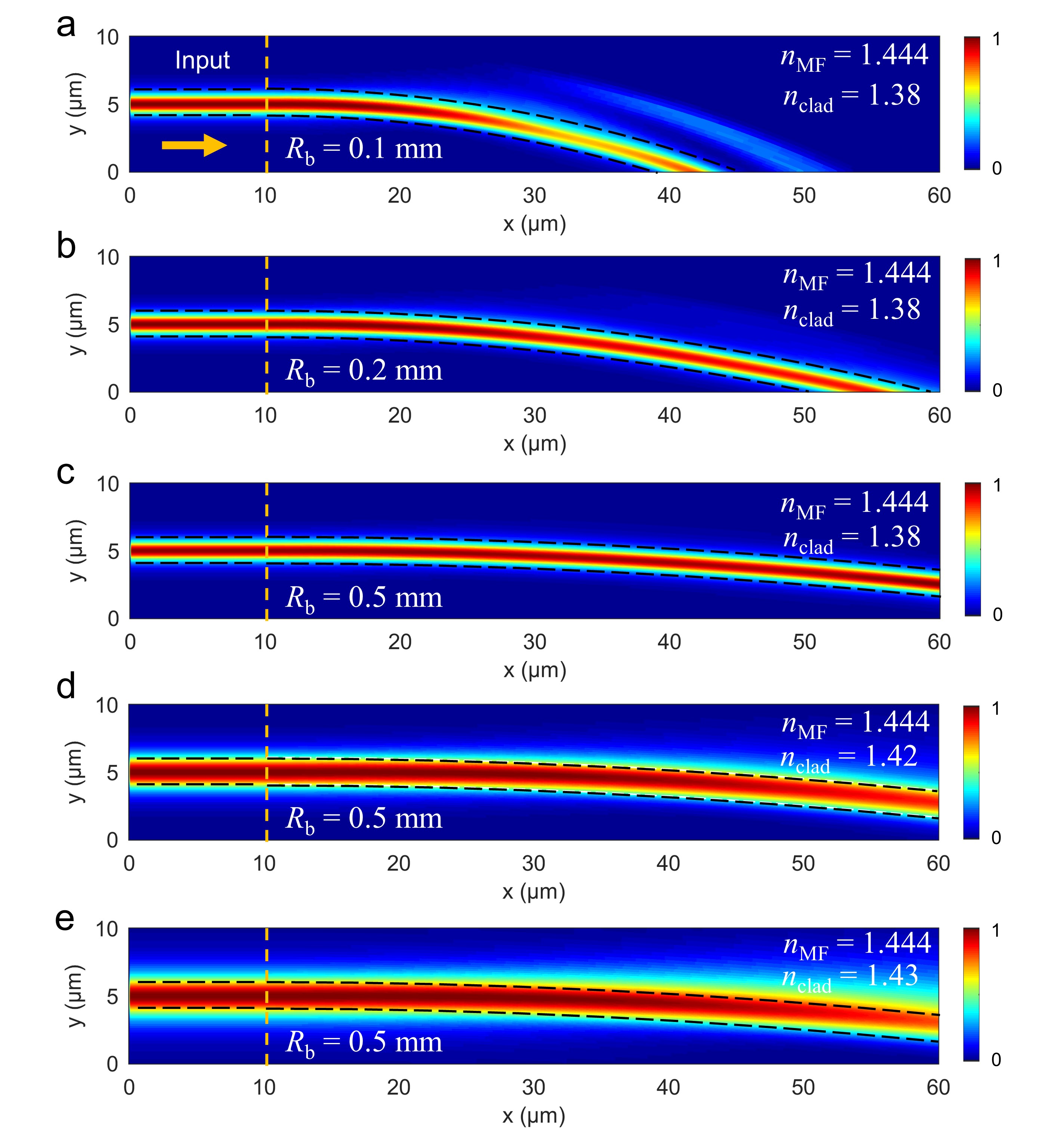
**

**Figure S2.** Simulations of field distribution in U-shaped MNFs with (a) *n*_MF_ = 1.444, *n*_clad_ = 1.38, *R*_b_ = 0.1 mm; (b) *n*_MF_ = 1.444, *n*_clad_ = 1.38, *R*_b_ = 0.2 mm; (c) *n*_MF_ = 1.444, *n*_clad_ = 1.38, *R*_b_ = 0.5 mm; (d) *n*_MF_ = 1.444, *n*_clad_ = 1.42, *R*_b_ = 0.5 mm; (e) *n*_MF_ = 1.444, *n*_clad_ = 1.43, *R*_b_ = 0.5 mm.

# Structural mechanics simulations

Figure S3a illustrates the correlation between the indenter’s downward displacement and the resulting normal force exerted on the protrusion. Longitudinal extrusions of several hundred micrometers can generate a normal force on the protrusion, reaching up to several newtons. Accordingly, insets in Figure S3a display deformation and pressure distribution of the protrusion under varying normal forces. With increasing normal force, the protrusion gradually flattens while maintaining its circular symmetry.

In addition to normal force, static friction between the indenter and the protrusion generates shear force on the protrusion. Lateral displacement of the indenter induces shear deformation in the protrusion. Figure S3b shows a linear correlation between the shear force along the +*x* direction and the lateral displacement induced by *x*-directional static friction. Insets in Figure S3b present the deformation and pressure distribution of the protrusion, where the *x*-directional shear deformation is clearly visible due to the asymmetric force applied. Notably, the shear forces along the *x*- and *y-*directions are independent of each other. The circular symmetry of the protrusion ensures that the y-directional shear force also adheres to a consistent response curve.


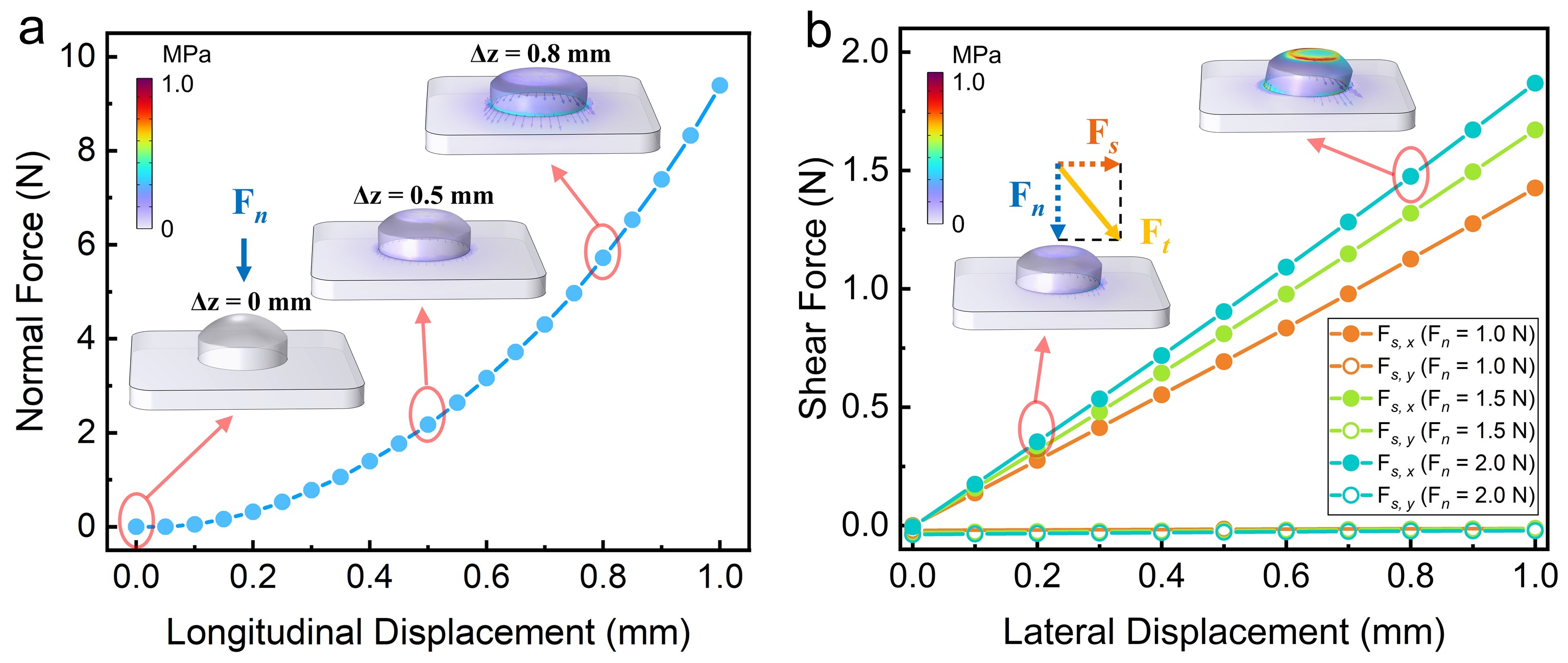


**Figure S3.** Structural mechanics simulations of PDMS protrusion. (a) The relationship between the indenter’s longitudinal displacement and the applied normal force on the protrusion. Insets: illustrations of the protrusion’s deformation and pressure distribution under varying normal forces. (b) The relationship between the *x*-directional shear force and the protrusion’s lateral displacement under varying normal forces. Insets: illustrations of the protrusion’s deformation and pressure distribution under varying shear forces along the *x*-direction.

Due to the photoelastic effect of the PDMS film, a reduction in the cladding refractive index results in increased bending loss for the U-shaped MNF (see also Figure S1d), contributing to its sensitivity to external forces. In the structural mechanics simulation shown in Figure S4, the photoelastic coefficient of the PDMS was set at 13.5×10^-5^/MPa^[3]^. When subjected to external forces, the PDMS elastomer exhibits an obvious change in refractive index, significantly affecting the transmission of the embedded MNF.

**
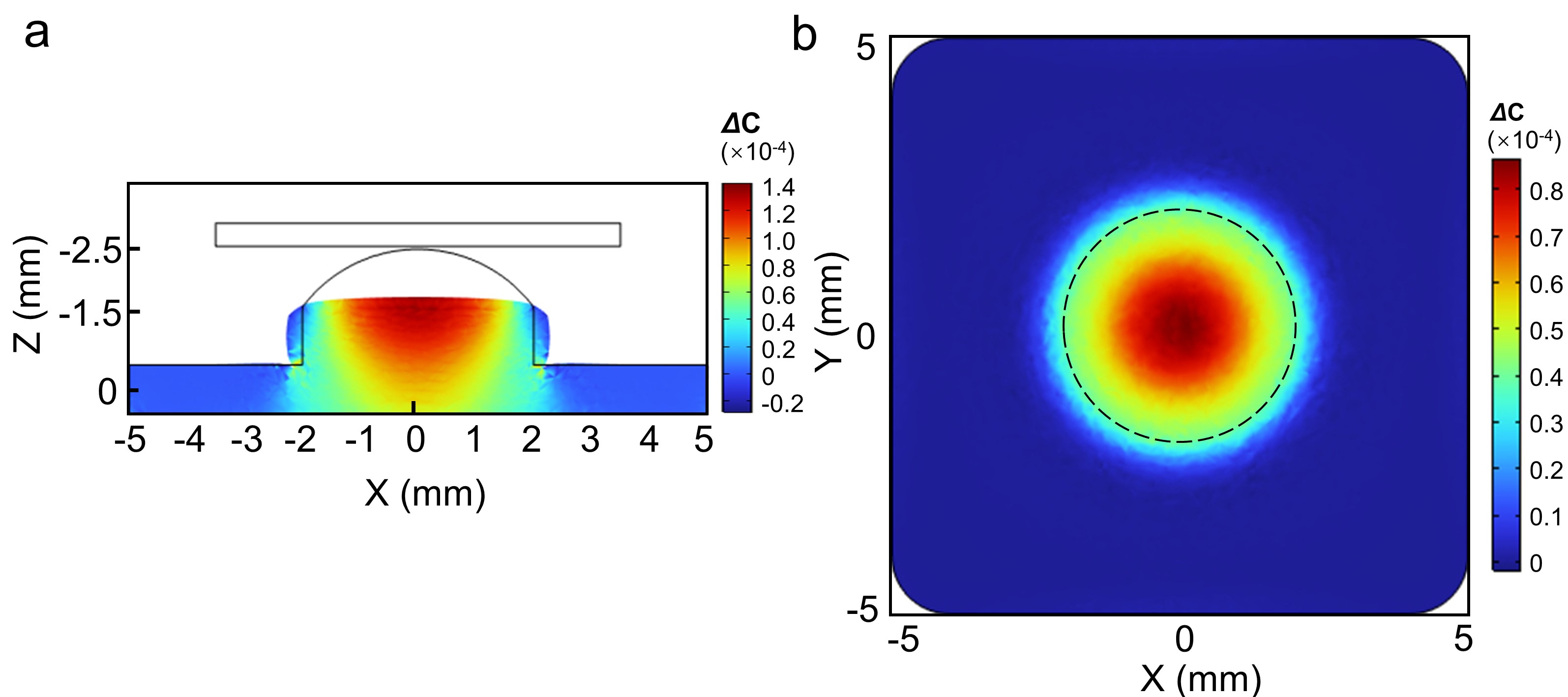
**

**Figure S4.** Simulation of photoelastic effect of the PDMS elastomer. Refractive index changes of (a) a longitudinal section and (b) the upper surface of the PDMS elastomer. The applied normal force is 9.3 N.

# Fabrication of MNFs

As depicted in Figure S5, during the fabrication of a SiO_2_ MNF, a significant multimode interference phenomenon initially occurs and then gradually diminishes, ending with an abrupt transmission drop due to the cutoff of high-order modes^[4]^. At this point, the diameter of fiber waist (i.e., MNF) is precisely 2 μm, with the total stretching length of fiber at approximately 22 mm. The diameter distribution of tapered fiber along its axial direction is described by the exponential attenuation formula, *D*(*Z*) = *D*_0_e^-^*^Z^*^/2.65(mm)^, where *D*(*Z*) denotes the axial diameter, *D*_0_ is the initial diameter (125 μm), and *Z* represents the one-sided drawing displacement. This technique ensures the reproducible fabrication of high-quality SiO_2_ MNFs. The resultant MNFs are inherently connected to standard optical fibers, offering benefits such as minimal transmission loss, compactness, and ease of integration.

**
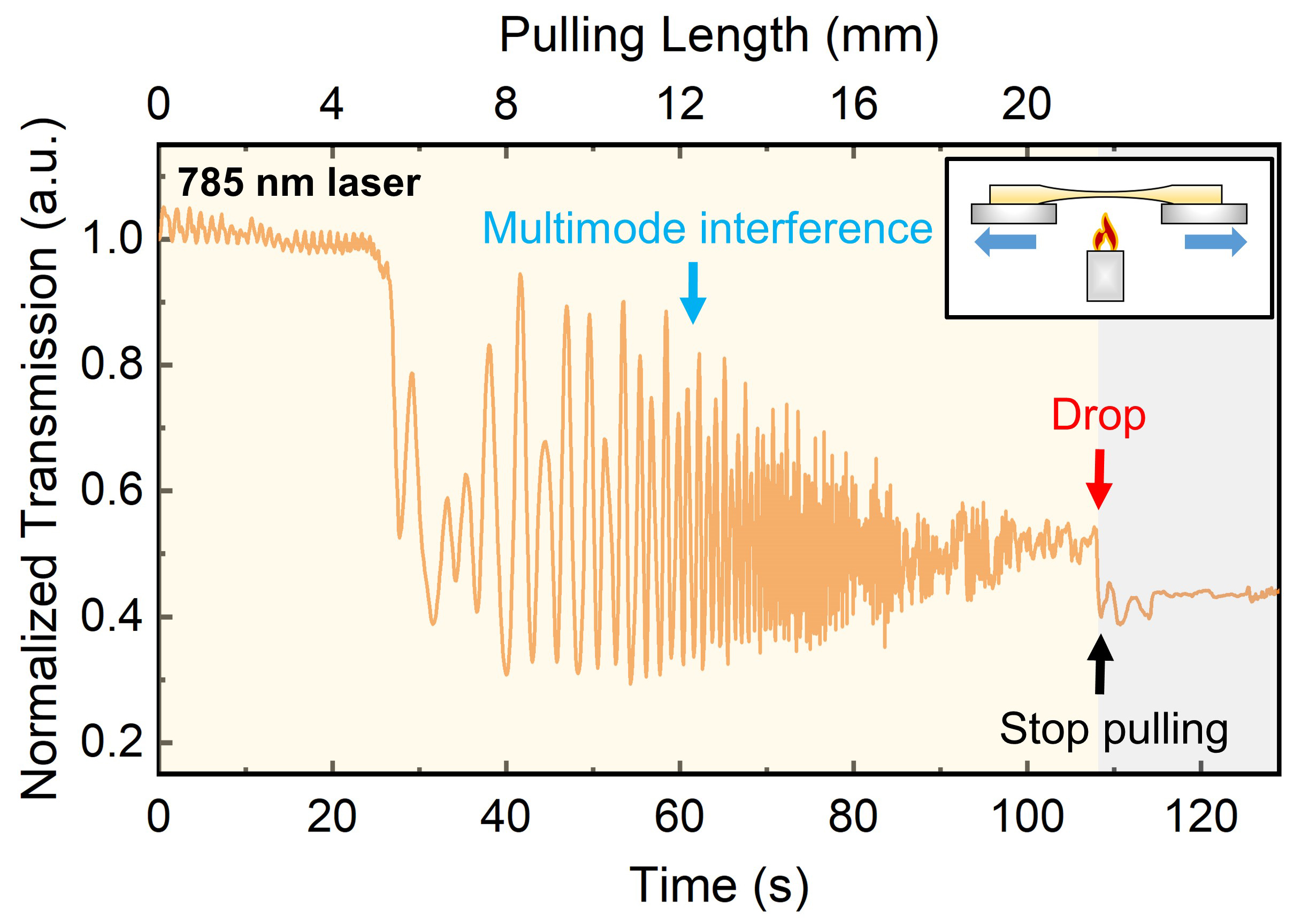
**

**Figure S5.** Normalized transmission of 785-nm laser throughout the taper-drawing process. Inset: schematic illustration of the taper-drawing technique.

# Fabrication of MNF sensors


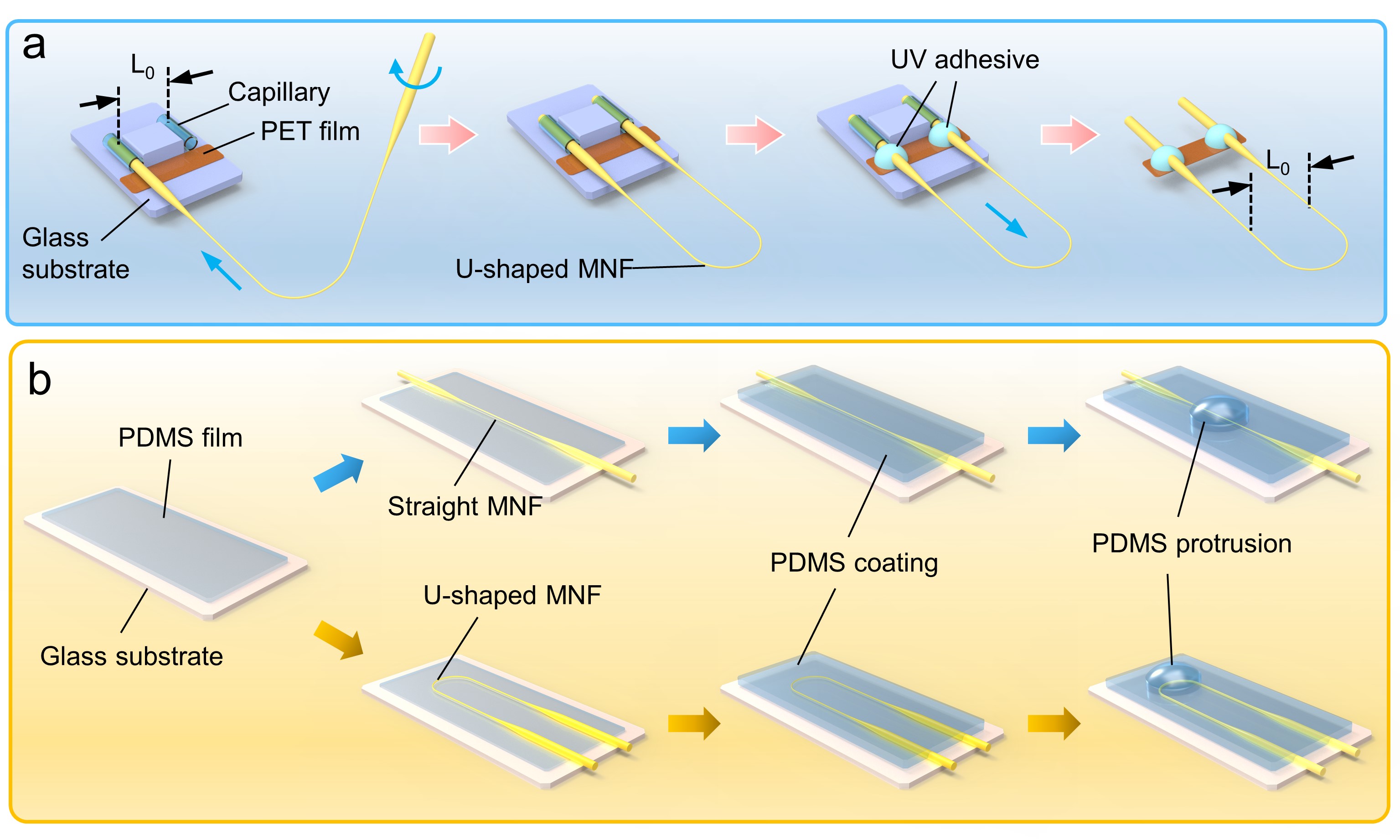


**Figure S6.** Schematic illustrations of fabrication processes for: (a) U-shaped MNF; (b) straight and U-shaped MNF sensors showing in the upper and lower panel, respectively.

# Response of sensors to normal force

**
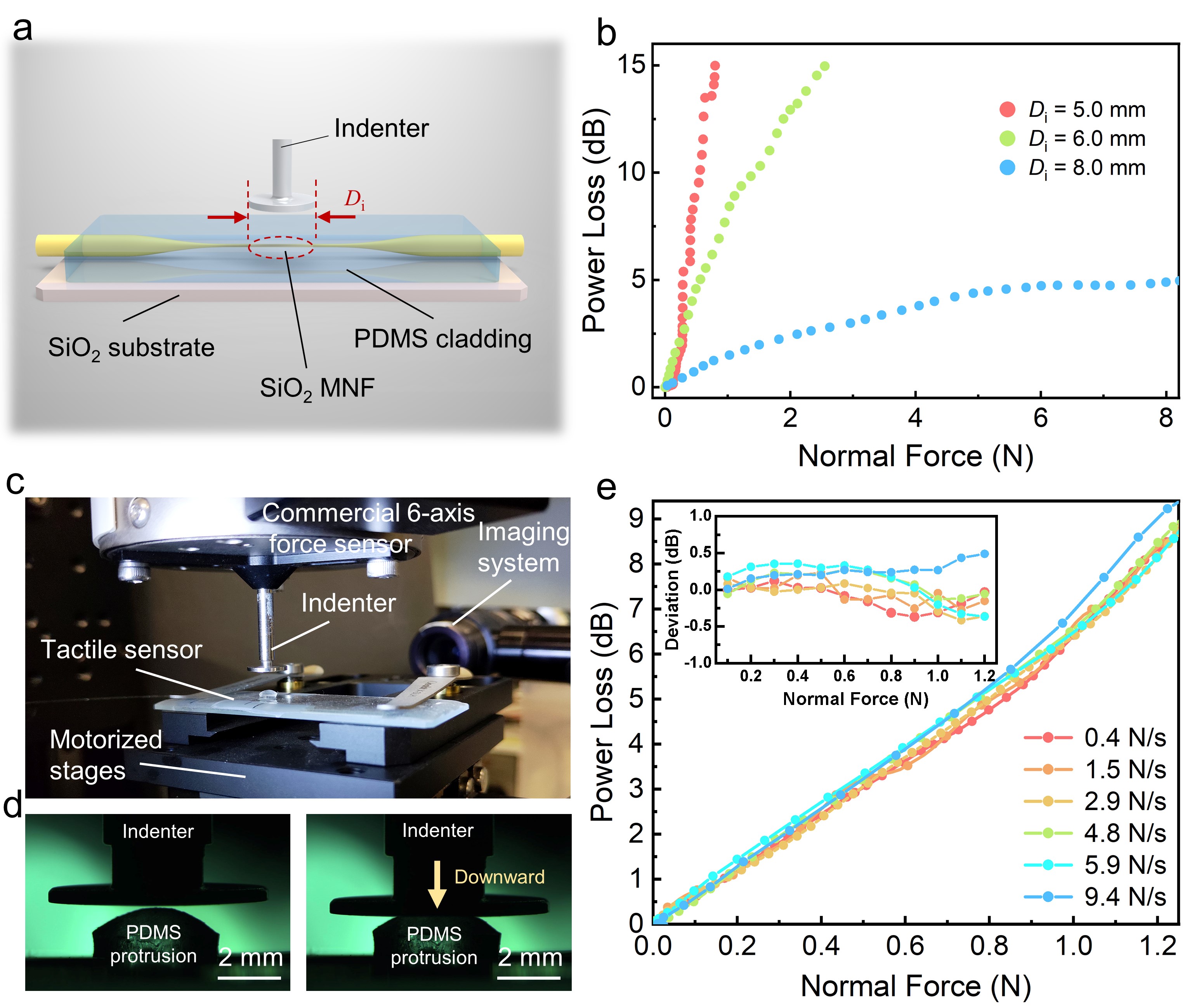
**

**Figure S7.** Response of sensors to normal force. (a) Schematic illustration for characterizing the response of a straight MNF-based sensor without the protrusion, where *D*_i_ denotes the diameter of indenter. (b) Normal force response of a straight MNF-based sensor without the protrusion. (c) Experimental setup for multiaxial force analysis. (d) Optical micrographs of the stainless-steel indenter aligned with the PDMS protrusion of a sensor. (e) Optical response of a U-shaped MNF sensor to normal forces at varying velocities. Inset: deviations from the average force value at different velocities.

# Multimode interference in PDMS-embedded MNF sensor

For a sensor fabricated from a PDMS-embedded MNF with a 2-μm diameter, when operating within the visible wavelength spectrum (e.g., input light wavelength of 600 nm, *n*_MF_ = 1.458, *n*_clad_ = 1.395), the *V*-number is calculated as *V* = π*D*_MF_/*λ*·(*n*_MF_^2^ – *n*_clad_^2^)^1/2^ = 4.6. Within the tapered fiber, multiple optical modes are intrinsically present, and mode coupling is likely to occur^[5]^. Typically, the MNF experiences increased bending loss when compressed. Simultaneously, an increase in the cladding refractive index modifies the coupling conditions of the optical modes, resulting in significant interference phenomena within the transmission spectrum (refer to Figures S8a,b). Mode coupling at a specific wavelength triggers a non-monotonic sensor response, significantly impairing the performance and practical application of sensor. To obtain a monotonic response, it is preferable to operate the MNF under the single-mode condition (*V* < 2.405), achievable by either reducing the fiber diameter or using a longer optical wavelength. Since narrowing the fiber diameter complicates the fabrication process of sensor, we circumvented this by operating the sensor at a wavelength of 1550 nm (*V* = 1.72 within the MNF). Additionally, the tapered fiber provided a graduated diameter distribution along its axis, which was simultaneously compressed by the protrusion. Even when the single-mode condition is met near the fiber waist, sections with wider diameters may cause dynamic multimode coupling when compressed, yielding non-monotonic output signal. As can be seen in Figure 2h, the transmissions of the straight MNF-based sensor exhibit non-monotonic behaviors (e.g., *D*_p_ = 4.5, 6.0 mm). However, the interference can be mitigated by reducing the protrusion diameter (e.g., *D*_p_ = 3 mm). As a result, a protrusion with a diameter of 4 mm was chosen for further experiments. Figures S8c,d show that under varying normal forces, sensors with either straight or U-shaped MNFs exhibit minimal multimode interference in their transmission spectra.

**
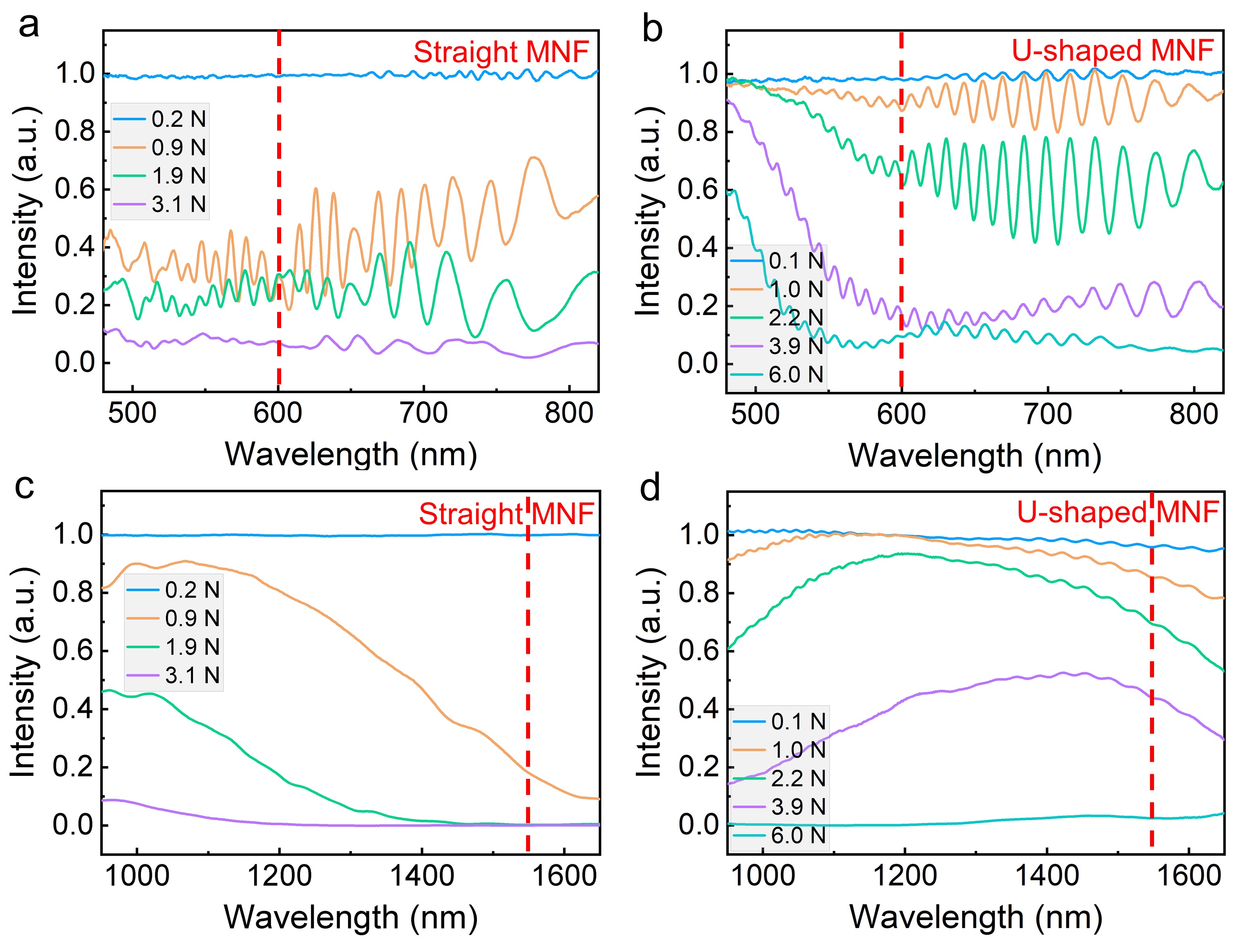
**

**Figure S8.** Normalized transmission spectra of the MNF sensors under varying normal forces. (a, b) Normal force-related transmission spectra of (a) the straight MNF and (b) the U-shaped MNF sensor within the visible wavelength region. (c, d) Normal force-related transmission spectra of the same (c) straight MNF and (d) U-shaped MNF sensor within the near-infrared region. The protrusion diameters of these sensors were approximately 4 mm.

# Overload test of U-shaped MNF sensor

**
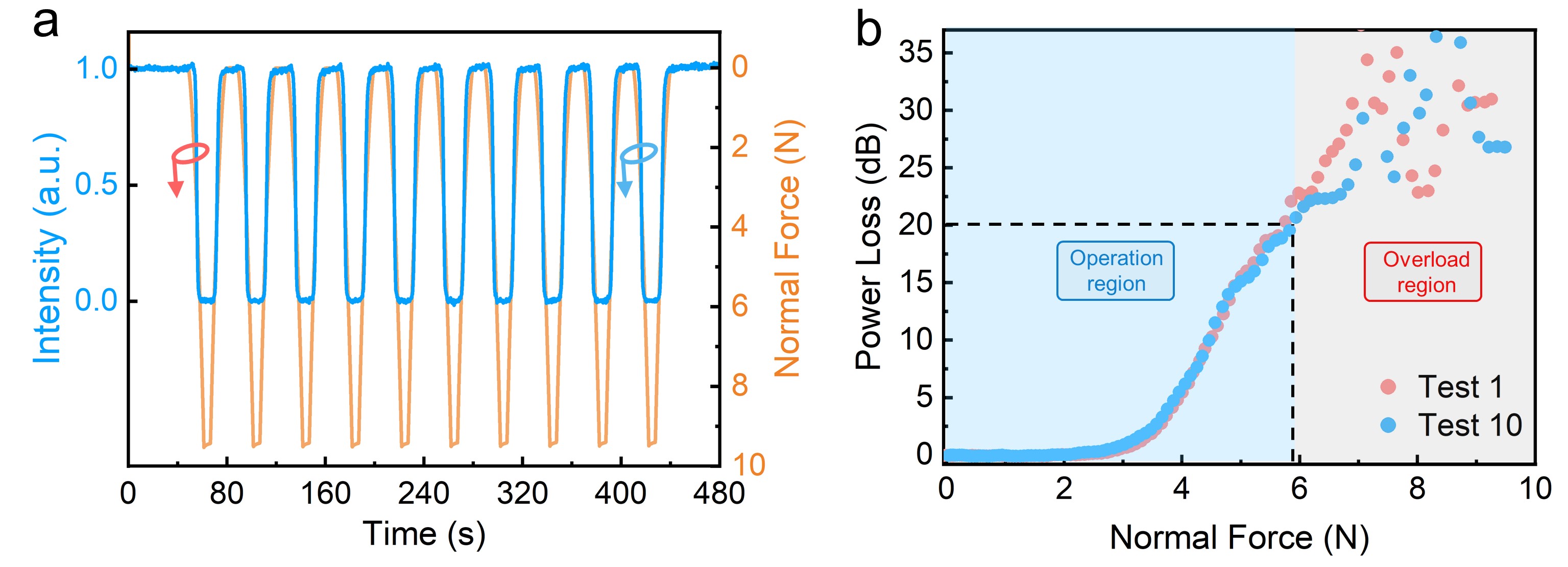
**

**Figure S9.** Overload test of the U-shaped MNF sensor. (a) Cyclic overload tests. (b) Response curve extracted from the initial and final cycles. The protrusion was at *x* ≈ +5 mm.

# Texture recognition


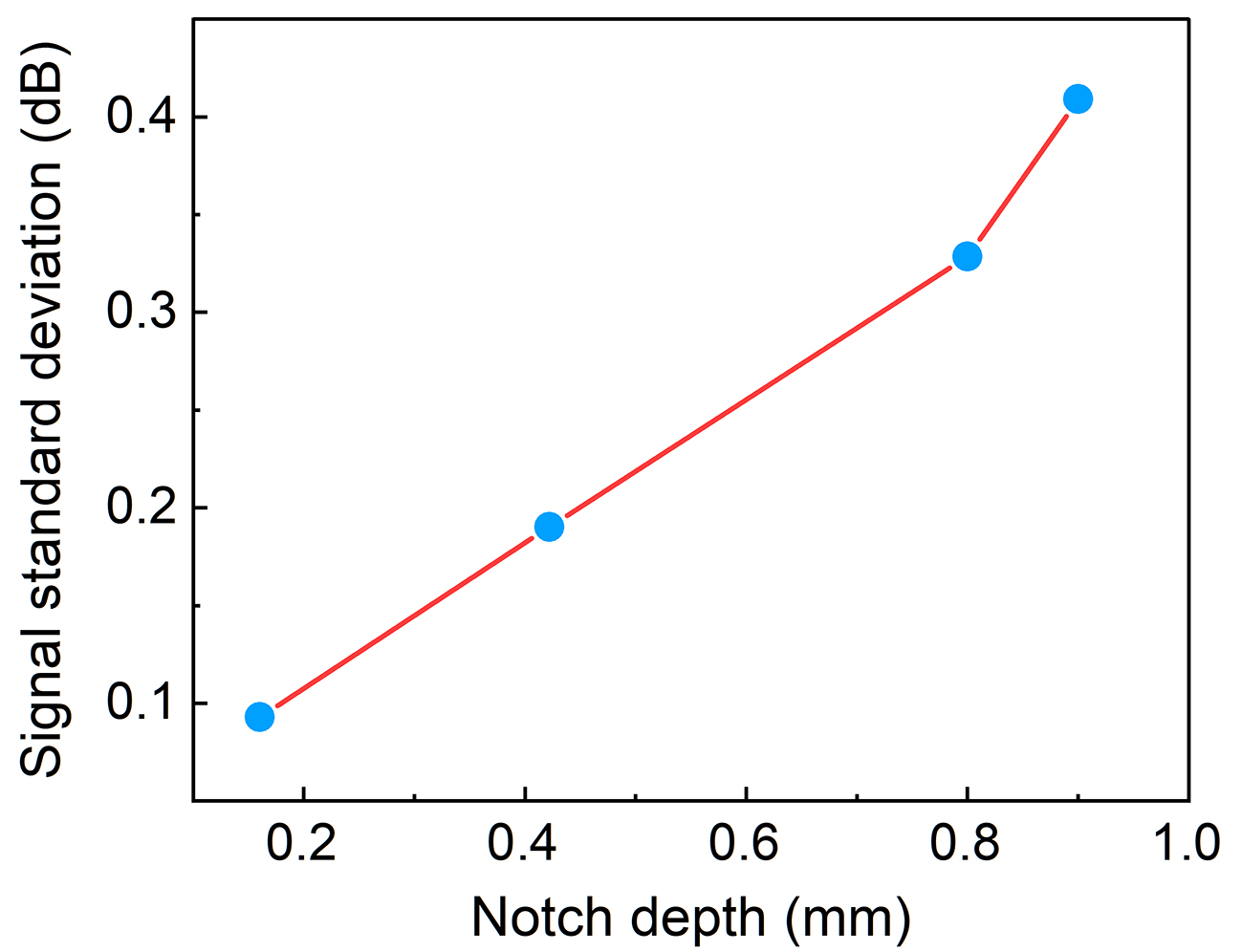


**Figure S10.** Signal standard deviation of texture recognition achieved by sliding the sensor over gratings with diverse periods and notch depths. The signal standard deviation was calculated from the transmission fluctuations depicted in Figure 5b.

**
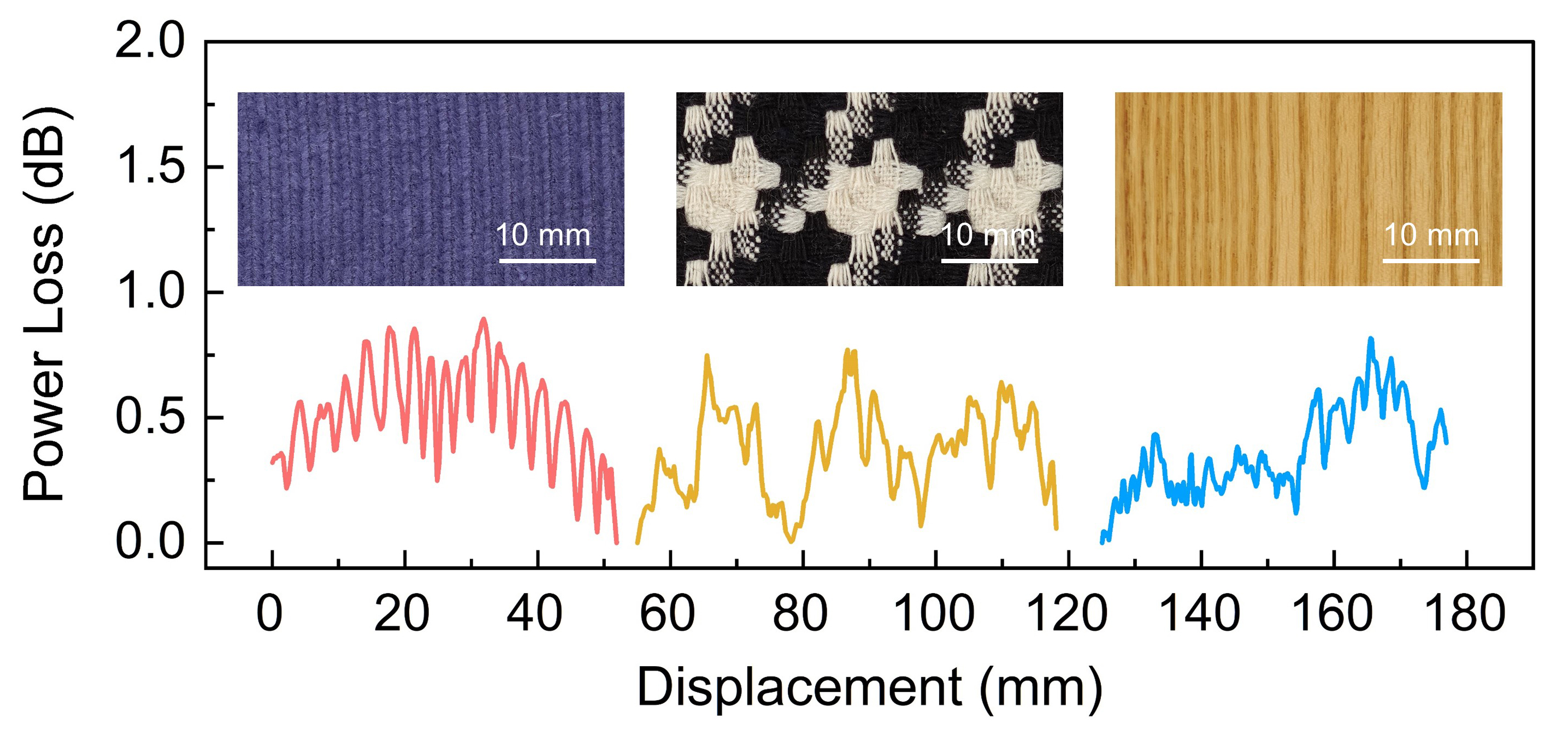
**

**Figure S11.** Texture recognition of three distinct specimens. The specimens arranged from left to right are corduroy fabric, tweed fabric, and plank, respectively.

**
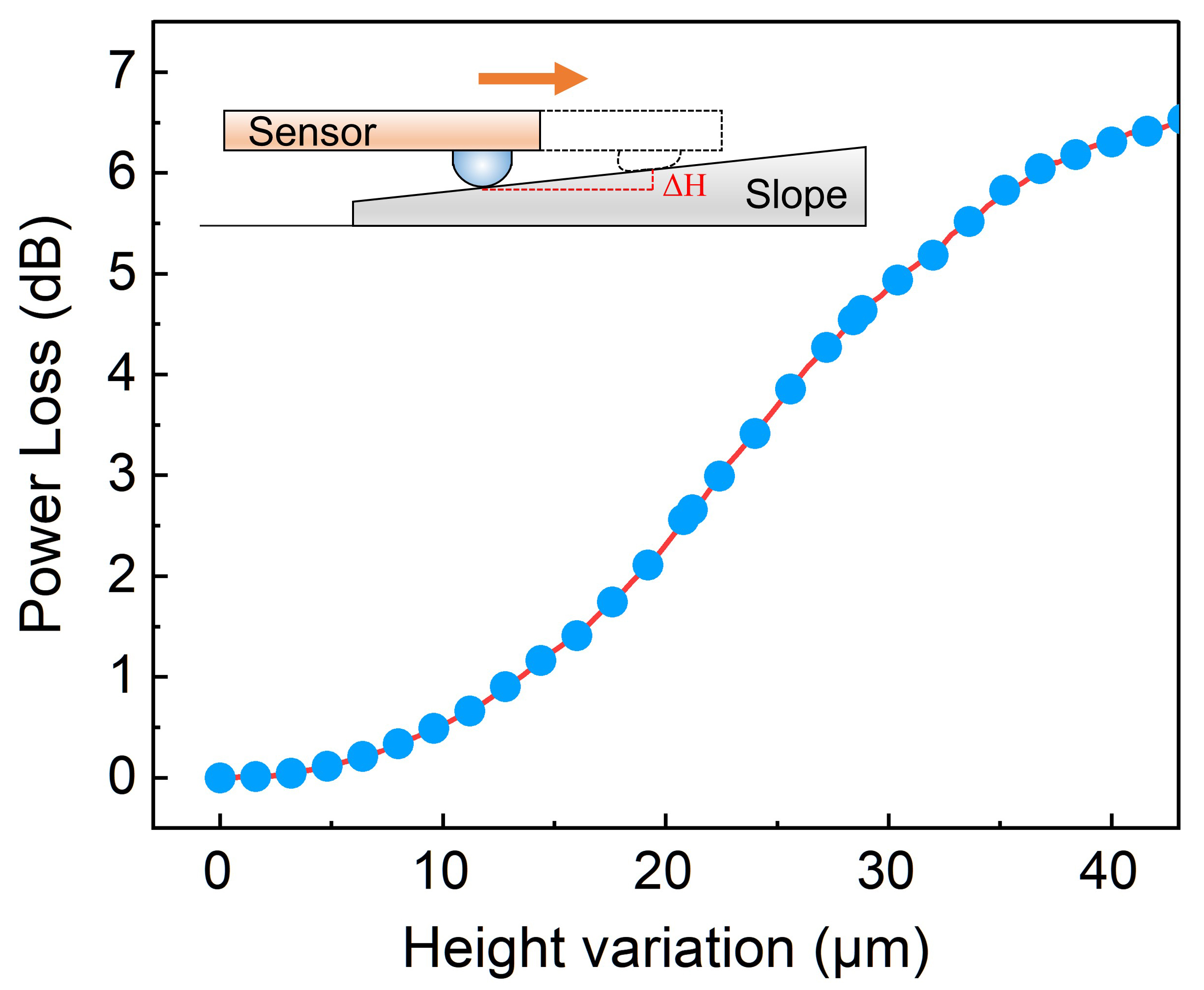
**

**Figure S12.** Correlation between sensor transmission and extrusion depth (i.e., ΔH) as it slid horizontally over a slope. Inset: illustration of the sensor sliding horizontally on the slope.

# Table S1. Comparison of optical multiaxial force sensors.

| **Reference (year)** | **Structural feature** | **Directional response** | **Performance** | | | | | **Application** | | | |
| --- | --- | --- | --- | --- | --- | --- | --- | --- | --- | --- | --- |
|  |  |  | ***Sensitivity*** | | ***Operation range*** | ***Force resolution*** | ***Overload resistance*** | ***Robotic grasping*** | ***Texture recognition*** | ***Tactile visualization*** | ***Human-machine interaction*** |
|  |  |  | ***Normal force*** | ***Shear force*** |  |  |  |  |  |  |  |
| Deng et al.^[6]^ (2020) | Fiber Bragg grating | **/** | 71 pm/N | ≤160 pm/N | 0-4 N | 6 mN | **/** | **/** | **/** | **/** | **/** |
| Cao et al.^[7]^ (2022) | Polymer waveguide network | **/** | < 0.97 dB/N | < 0.97 dB/N | 0-1.1 N | 0.1 mN | **/** | **/** | **/** | **/** | **/** |
| Zhou et al.^[8]^ (2022) | Crossed over polymer waveguides | Partially | ~7.3 dB/N | ~3.6 dB/N | 0-1 N | 0.52 mN | 4.5 times | **○** | **/** | **/** | **/** |
| Pan et al.^[9]^ (2023) | Polymer fiber knot | **○** | ~0.27/N (0.012 dB/N) | ~0.056/N (0.002dB/N, frictional force) | 0-20 N | **/** | **/** | **○** | **/** | **/** | **/** |
| Chen et al.^[10]^ (2023) | Two-layered weaved polymer fiber | **○** | < 2 dB/N | < 2 dB/N | 0-2 N | **/** | **/** | **/** | **/** | **/** | **/** |
| Leslie et al.^[11]^ (2023) | LEDs illuminated internal cavity | **/** | / | / | 0-2 N | ~20 mN | **/** | **○** | **/** | **/** | **/** |
| Gan et al.^[12]^ (2023) | Fiber Bragg grating | **/** | 140.5 pm/N | <500 pm/N | 0-1 N | > 1 mN | **/** | **/** | **/** | **/** | **/** |
| This work | U-shaped MNF | ○ | 50.7 dB/N (14%/kPa) | 82.2 dB/N (21%/kPa) | 0-0.8 N | 120 μN | 3.8 times | / | ○ | ○ | ○ |

Notes: “**○**” denotes reported, “/” denotes not reported.

# References

[1] L. M. Tong, J. Y. Lou, E. Mazur, *Opt. Express* **2004**, *12*, 1025.

[2] X. Q. Wu, L. M. Tong, *Nanophotonics* **2013**, *2*, 407.

[3] D. W. Van Krevelen, K. Te Nijenhuis, Elsevier, Netherlands **2009**.

[4] Y. X. Xu, W. Fang, L. M. Tong, *Opt. Express* **2017**, *25*, 10434.

[5] P. F. Wang, H. Y. Zhao, X. F. Wang, G. Farrell, G. Brambilla, *Sensors* **2018**, *18*, 858.

[6] Y. Deng, T. Yang, S. Dai, G. Song, *IEEE Trans. Biomed. Eng.* **2020**, *68*, 2339.

[7] D. Cao, J. Hu, Y. Li, S. Wang, H. Liu, *IEEE Robot. Autom. Lett.* **2022**, *7*, 3443.

[8] J. Zhou, Q. Shao, C. Tang, F. Qiao, T. Lu, X. Li, X. J. Liu, H. Zhao, *Adv. Mater. Technol.* **2022**, *7*, 2200595.

[9] J. Pan, Q. Wang, S. Gao, Z. Zhang, Y. Xie, L. Yu, L. Zhang, *Opto-Electron. Adv.* **2023**, *6*, 230076.

[10] W. Chen, Y. Yan, Z. Zhang, L. Yang, J. Pan, "Polymer-based self-calibrated optical fiber tactile sensor", presented at *2023 IEEE/RSJ International Conference on Intelligent Robots and Systems (IROS)*, 2023.

[11] O. Leslie, D. Córdova Bulens, S. J. Redmond, *Sensors* **2023**, *23*, 9640.

[12] L. Gan, J. Wang, L. Xie, Y. Zhou, *IEEE Trans. Instrum. Meas.* **2023**, *73*, 7001511.
